# Impact of Childhood Obesity and Psychological Factors on Sleep

**DOI:** 10.3389/fpsyt.2021.657322

**Published:** 2021-07-09

**Authors:** Nazar Mazurak, Jessica Cook, Alisa Weiland, Yvonne Ritze, Michael Urschitz, Florian Junne, Stephan Zipfel, Paul Enck, Isabelle Mack

**Affiliations:** ^1^Department of Psychosomatic Medicine and Psychotherapy, University Hospital, Tübingen, Germany; ^2^Institute of Medical Psychology and Behavioral Neurobiology, University Hospital, Tübingen, Germany; ^3^Division of Paediatric Epidemiology, Institute of Medical Biostatistics, Epidemiology, and Informatics, University Medical Centre of the Johannes Gutenberg-University, Mainz, Germany

**Keywords:** childhood obesity, sleep, prevention, psychology, inpatient treatment

## Abstract

The aim of the study was to analyze sleep duration and behaviors in relation to psychological parameters in children and adolescents with obesity seeking inpatient weight-loss treatment in comparison to normal-weight children, and whether or not these variables would improve during the time course of treatment. Sixty children or adolescents with overweight and obesity (OBE) and 27 normal-weight (NW) peers (age: 9–17) were assessed for subjective sleep measures through self-reported and parent-reported questionnaires, as well as body weight, body composition, and psychological questionnaires. The OBE participants were assessed upon admission and before discharge of an inpatient multidisciplinary weight-loss program. NW participants' data were collected for cross-sectional comparison. In comparison to NW, children and adolescents with OBE had a shorter self-reported sleep duration and had poorer sleep behaviors and more sleep-disordered breathing as reported by their parents. No change in sleep measures occurred during the inpatient treatment. Psychological factors including higher anxiety, depression, and destructive-anger-related emotion regulation were moderate predictors for unfavorable sleep outcomes, independent of weight status. Children with obesity had less favorable sleep patterns, and psychological factors influenced sleep in children, independent of weight. More research is needed on the relationship and direction of influence between sleep, psychological factors, and obesity, and whether they can be integrated in the prevention and management of childhood obesity and possibly also other pediatric diseases.

## Introduction

Obesity has been declared a global epidemic by the World Health Organization, with incidents of overweight and obesity increasing globally and occurring earlier in the lifespan (1, 2). The consequences of childhood obesity are far reaching, impacting physical, emotional, and social wellbeing, as well as short-term and long-term comorbidities including cardiovascular, metabolic, respiratory, and orthopedic conditions (3). Due to this, there is significant focus worldwide on the prevention and treatment of obesity in children and adolescents. Obesity is a multifactorial condition with a complex etiology, and disordered sleep patterns appear to be contributing to the complex interplay (3–5). Disordered sleep and short sleep duration have been found to be associated with obesity in studies in adult and child and adolescent populations (6–8).

The National Sleep Foundation recommends an optimal sleep duration of 10 to 13 h for children aged 3 to 5 years old, 9 to 11 h for children aged 6 to 13 years, and 8 to 10 h for adolescents aged 14 to 17 years (9, 10). However, most children and adolescents do not achieve these sleep durations (11). Additionally, the prevalence of sleep disorders is reported at between 20% and 45% in children and adolescents (12).

The relationship between sleep and weight status in children and adolescents has been a growing focus in research over the last decade, with studies exploring various parameters including sleep duration, weekday–weekend variability, sleep behaviors, and sleep disorders. The exact mechanism behind the relationship between undesirable sleep patterns and weight dysregulation remains unclear, with proposed mechanisms including changes in dietary intake, media use, and physical activity resulting in greater exposure to the obesogenic environment, longer eating window, reduced eating-related inhibitory control, increased reward sensitivity, and changes in hormonal regulation of hunger and satiety (13, 14). In children and adolescents, higher BMI z-score and waist circumference have been associated with poorer sleep quality and behaviors (4, 15, 16), and higher body fat percentage has been associated with later average bed times (16). The relationship between average sleep duration over the week and childhood obesity has slightly mixed findings (6, 14, 17, 18); however, shorter sleep duration on school days specifically has been associated with higher BMI z-scores (19) and increased prevalence of eating in the absence of hunger (14). It is further evident in comparisons between children and adolescents receiving weight-loss treatment and their normal-weight peers, with studies finding that 88% of children participating in a weight-loss program sleep less than recommended (20) and that inpatient weight-loss program participants have shorter and more disturbed sleep than controls (4). However, research into the sleep patterns of pediatric patients in an inpatient setting is rarely conducted in relation to childhood obesity.

In addition to obesity being associated with poorer sleep measures, obesity in children and adolescents has also been associated with significantly higher psychological and social burden (20, 21). An increased prevalence of psychological symptoms related to depression and anxiety disorders has been found in children and adolescents with obesity (22, 23). In addition, associations have been identified for social and behavioral concerns, including increased risk of social isolation and poorer peer relationships, discrimination, harassment, and poor self-esteem, in comparison to their normal-weight peers (21, 24–26). These factors can worsen the quality of life of the child or adolescent with obesity (3), with the potential to have long-lasting implications, as obesity in childhood often leads to obesity in adulthood (2, 3, 5).

The aim of this study was to analyze sleep behavior in relation to weight status in children and adolescents with obesity seeking inpatient weight-loss treatment, and whether or not these variables would improve during the time course of treatment. In a cross-sectional approach, we compared the baseline sleep measures of this group to that of a normal-weight comparison group, matched for age and sex, and investigated the relationship between sleep and psychological factors such as anxiety, stress, and depression.

Therefore the following hypotheses were proposed:

1. Children and adolescents with overweight and obesity have shorter sleep duration and less favorable sleep behaviors at baseline than those with normal weight.

2. The sleep duration and sleep behaviors of the children and adolescents with overweight and obesity improve over the course of the inpatient treatment, and these changes will be associated with improvements in BMI z-score and body fat percentage.

3. Unfavorable sleep behaviors in children and adolescents are related to psychological factors (anxiety, stress, and depression), quality of life, and weight status.

## Materials and Methods

### Study Design and Participants

The present study was conducted as part of the DROMLIN study (PreDictor Research in Obesity during Medical care weight-loss in children and adolescents during an INpatient rehabilitation) (27) and is registered at the German Clinical Trials Register (DRKS) with the clinical trial number DRKS00005122. The study protocol was approved by the Ethics Committee of the medical faculty for the University Tübingen, Germany. The study was conducted in 2012–2013, and the children and parents were informed about the purpose of the study and provided written informed consent prior to commencement.

Sixty children with overweight or obesity [OBE; BMI percentile ≥90 (28)] aged 9 to 17 years (47% males) with an indication for inpatient weight-loss intervention were included. All OBE took part in an inpatient weight-loss program at the Children Rehabilitation Hospital for Respiratory Diseases, Allergies and Psychosomatics in Wangen i.A., Germany. The program followed a multidisciplinary approach in accordance with the latest developments in medicine and operated in close cooperation with regional educational institutions, such as the Obesity Academy in Baden-Württemberg (Adipositas Akademie Baden-Württemberg e.V.). Children were treated in small therapeutic groups with peers of the same age and housed in residential units situated on a park-like hospital ground. The hospital had its own school with regular classes for all types of curricula. The program comprised physical activity, cognitive behavioral therapy, and a balanced diet. Smoking was forbidden during the inpatient stay. Exclusion criteria were severe psychological comorbidities, linguistic or intellectual limitations, type-1 diabetes, malignant tumors, systemic disorders, or severe cardiovascular diseases. The full details regarding inclusion and exclusion criteria and the conducted therapy are detailed elsewhere (27). Additionally, 27 normal-weight (NW; BMI percentile <90) children and adolescents matched for sex and age as a comparison group were recruited from the surrounding area of the University Hospital Tübingen, Germany. The advertising was performed *via* email for university employees and local newspaper. The children received a voucher for a book in a local bookstore (15 Euros) as incentive.

Baseline measures (T0) for the OBE group were conducted in the first week of the inpatient therapy over two consecutive days. Parents also completed questionnaires relating to their child's sleep and psychological factors at T0. Time point T1 occurred in the last week of inpatient therapy and was conducted identically to T0. NW data were collected at a single time point at our laboratory for comparison and included anthropometric, sleep behavior, and psychological measures.

### Assessments

#### Anthropometry

Height and weight were measured onsite at baseline (T0) and upon discharge from treatment (T1) using standardized techniques. Participants' height and weight were converted to BMI, which were applied to the appropriate sex-specific BMI reference charts to determine BMI percentile and z-score at each time point (28). Percentage body fat was determined using a Lipometer (Möller Messtechnik, Graz, Austria) at 15 prespecified anatomical points, providing noninvasive, reliable, and valid measures (29, 30).

#### Sleep and Psychological Factors

Standardized and age-validated questionnaires were completed by the children and parents in both a cross-sectional (completed by OBE and NW and their parents at T0) and longitudinal design (completed at T0 and T1 by OBE). These questionnaires covered aspects of sleep duration and sleep behaviors, and psychological factors such as anxiety, stress and depression, self-esteem, and quality of life. The specific details of the questionnaires are summarized in Table 1.

**Table 1:** Questionnaires to assess sleep and psychological factors.

**Questionnaire**	**Short description**
**Questionnaires for children**	
Sleep self-report (SSR) (31)	28 items; three thematic areas, three answer options ranging from “common” to “uncommon”
Pediatric daytime sleepiness scale (PDSS) (32)	32 items; six thematic areas; five answer options ranging from “always” to “never”
Inventory for recording the quality of life in children and adolescents (ILK) version for children (33)	10 items; seven thematic areas; five answer options ranging from “very good” to “very bad”
Somatization inventory for children and adolescents (SI-KJ) (34)	35 items; list of symptoms; five answer options ranging from “not at all” to “much”
State-trait anxiety inventory for children (STAI-C) (35)	20 items; two scales; three answer options ranging from “almost never” to “often”
Depression inventory for children and adolescents (DIKJ) (36)	26 items; queries gradation of symptoms; decision between three predefined answer alternative
Stress and stress coping questionnaire for children and adolescents (SSJK) (37)	84 items; three subscales; five answer options ranging from “never” to “always”
Revised self-esteem scale by Rosenberg (38)	10 items; total score from 10 to 40, the higher the score the higher the level of self-esteem; four answer options ranging from “strongly disagree” to “strongly agree”
**Questionnaires for parents**	
Children's sleep habits questionnaire (CSHQ)(39)	33 items; eight subscales; three answer options ranging from “common” to “uncommon”
Pediatric sleep habits questionnaire (PSQ) (40)	22 items; three subscales; Answer options: Item 1 to 16: “yes”, “no”, “don't know”; item 17 to 22: four options ranging from “does not apply” to “meets most of time”
Epworth sleepiness scale (ESS-E) (41)	8 items; total score from 0 to 24; four answer options ranging from “would never doze” to “dozing high probability”
Strength and difficulties questionnaire (SDQ) (42)	25 items; five subscales; three answer options ranging from “does not apply” to “clearly applicable”
Inventory for recording the quality of life in children and adolescents (ILK) version for parents (33)	11 items; nine thematic areas; five answer options ranging from “very good” to “very bad”

The specific details of the questionnaires are summarized below.

##### Questionnaires for Children

*Sleep Self-Report.* This established, validated self-report questionnaire is a screening tool for assessing overall sleep problems. The development of the SSR was based on the Children's Sleep Habits Questionnaire (CSHQ). The SSR consists of 26 items with three answer options ranging from “common (5–7×/week)” to “uncommon (0–1×/week)”. The items are about sleep duration, bedtime resistance, night awakenings, and daytime sleepiness. A score of 23 items is calculated. For the German version, the internal consistency was acceptable with a Chronbach's α = 0.73 and a retest reliability of *r* = 0.58 (31). In this study population, Chronbach's α = 0.75.

*Pediatric Daytime Sleepiness Scale.* This validated questionnaire assesses daytime sleepiness and consists of eight items (32). The five answer options range from “never” to “always.” Internal consistency is good with Chronbach's α = 0.8. In this study population, Chronbach's α = 0.75.

*Inventory for Recording the Quality of life in Children and Adolescents Version for Children.* This validated tool assesses quality of life. It consists of 10 items and covers the thematic areas school, family, social contacts among peers, interests and free-time activities, physical health, and psychological health. A sum score is calculated for overall quality of life. The five answer options range from “very good” to “very bad.” Chronbach's α was between 0.55 and 0.76 and the test–retest reliability between *r* = 0.6 and 0.8 (33). In this study population, Chronbach's α = 0.72.

*Depression Inventory for Children and Adolescents.* This validated questionnaire is used to assess the severity of depressive symptoms according to Diagnostic and Statistical Manual of Mental Disorders (DSM−5) criteria. It consists of 26 items and three predefined answer alternatives, allowing for calculation of a sum score. Internal consistency of the German version was reported with Chronbach's α = 0.87 to 0.92 (36). In this study population, Chronbach's α = 0.88.

*Somatization Inventory for Children and Adolescents.* This validated questionnaire provides information about intensity, frequency, and types of somatic complaints in the areas pseudoneurological symptoms, cardiovascular symptoms, gastrointestinal symptoms, and pain (34). It consists of 35 items and has five answer options ranging from “not at all” to “much”. The internal consistency was high with Chronbach's α = 0.93 (43). In this study population, Chronbach's α = 0.90.

*State-Trait Anxiety Inventory for Children.* The established, validated questionnaire measures state anxiety (anxiety about an event) and trait anxiety (anxiety as a personal characteristic). In this study, trait anxiety was assessed, consisting of 20 items and allowing for calculation of a final sum score. The three answer options range from “almost never” to “often.” Internal consistency of the German version was reported with Chronbach's α = 0.81 and test–retest reliability with *r* = 0.64 (35). In this study population, Chronbach's α = 0.90.

*Stress and Stress Coping Questionnaire for Children and Adolescents.* This validated tool assesses the degree of self-reported stress and coping with stress. It consists of 84 items covering the subscales stress vulnerability, stress cooping, and stress symptoms. The five answer options range from “never” to “always.” Values for internal consistency (Chronbach's α) are between 0.67 and 0.89 and test–retest reliability between *r* = 0.56 and 0.82 (37). In this study population Chronbach's α ranges between the subscales from 0.69 to 0.87.

*Self-esteem Scale by Rosenberg.* This validated questionnaire assesses self-esteem using 10 items with subsequent calculation of a sum score. The four answer options range from “strongly disagree” to “strongly agree.” In the German version, the internal consistency Chronbach's α ranges between 0.72 and 0.85 (38). In this study population, Chronbach's α = 0.90.

##### Questionnaires for Parents

*CSHQ.* This questionnaire is an established, validated parents' questionnaire for assessing sleep problems of their children. It consists of 33 items with three answer options ranging from “common (5–7×/week)” to “uncommon (0–1×/week).” A total score of 33 items and 8 subscores comprising the areas bedtime resistance, sleep-onset delay, sleep duration, sleep anxiety, night wakings, parasomnias, sleep-disordered breathing, and daytime sleepiness are calculated. In the German version, the internal consistency Chronbach's α = 0.68, and test–retest reliability of *r* = 0.76 (39, 44). In this study population, Chronbach's α = 0.78.

*Epworth Sleepiness Scale.* This validated parents' questionnaire is used to assess daytime sleepiness in their children (45). The development was based on the ESS version for adults with Chronbach's α = 0.88 and test–retest reliability of *r* = 0.82 (41, 46). It consists of eight items and four answer options ranging from “would never doze” to “dozing high probability”. Finally, a sum score is calculated. In this study population, Chronbach's α = 0.73.

*Pediatric Sleep Habits Questionnaire.* This validated parents' questionnaire is used to assess sleep-disordered breathing, snoring, sleepiness, and behavioral problems of their children. It consists of 22 items and the three subscales snoring, sleepiness, and behavior. In addition, a total sum score is calculated. The answer options are “yes”, “no”, and “don't know”. For items 17 to 22, the four answer options range from “does not apply” to “meets most of time”. The internal consistency (Chronbach's α = 0.89) and test–retest reliability were considered as good (40). In the German study population, Chronbach's α = 0.72 (47). In this study, Chronbach's α = 0.73.

*Inventory for Recording the Quality of life in Children and Adolescents Version for Parents.* This validated parent's questionnaire assesses quality of life of their children and is a similar construct to the children/adolescents' version as described above but consists of 11 items (33). In this study, Chronbach's α = 0.62.

*Strength and Difficulties Questionnaire.* This validated parent's questionnaire is used to measure pro-social behavior, emotional symptoms, conduct problems, hyperactivity, and peer problems of their children. It consists of 25 items which allow calculation of the five subscales emotional symptoms, conduct problems, hyperactivity–inattention, peer relationship problems, and prosocial behaviors. A sum problem score is calculated including all items. The three answer options range from “does not apply” to “clearly applicable.” Internal consistence (Chronbach's α = 0.82) was considered as good for the German version (42, 48). In this study, Chronbach's α = 0.73.

In addition to the questionnaires, sleep duration was self-reported by the children at T0 with the question “*How many hours per night do you sleep on average?”* and at T1 with the question “*How many hours per night have you been sleeping on average while here in Wangen?”* The answers were recorded using a five-point scale (5 = 9–11 h, 4 = 8–9 h, 3 = 7–8 h, 2 = 5–7 h, 1 = <5 h). Sleep duration was also reported by the parents at baseline as a component of the CSHQ and was recorded to one decimal place (e.g., 8.5 h).

Sleep quality was self-reported by the children at T0 with the *question “How well do you sleep at home on average?”* and at T1 with the question “*How well have you slept on average during your inpatient stay?”* The answers were collected using a visual 10-point scale (0 = very good to 10 = very bad).

### Data Items and Statistics

Data analysis was completed using the Statistics software IBM SPSS 21 (SPSS Inc., Chicago, IL, USA). Differences in baseline measures for OBE and NW were analyzed *via* independent t-tests and Mann–Whitney-*U* tests, and differences in sleep and anthropometric measures between baseline (T0) and after therapy (T1) *via* paired t-tests and Wilcoxon signed-rank tests, depending on data distribution. Spearman's rank correlations were conducted to assess the relationship between changes in sleep outcomes and anthropometric changes. To assess statistical significance of group differences and correlations, all p-values were adjusted for multiple testing using the false discovery rate (FDR) method, with the alpha level set at FDR <0.05 (47).

Multiple linear regression modeling with child- and parent-reported sleep questionnaires (SSR, PDSS, CSHQ, ESS-E, and PSQ-SRBD) as dependent variables and STAI-C, DIKJ, SSKJ, and SDQ questionnaires as predictors was performed. Group (OBE vs. NW), age, gender, and initial BMI were also included in linear modeling. For the purpose of regression analysis, missing data were replaced with variables' means. Due to the small sample size, data were corrected for multiple comparison in the regression analyses. This was achieved by the preselection of independent variables that were included in each model. Therefore, first a Spearman correlation matrix for all dependent and independent variables was calculated. Second, the p-values were corrected using the FDR method. Finally, correlations which remained significant after step 2 were included in further regression modeling. The variable group was included in each regression analysis in order to explore the influence of weight status on the relations between sleep variables and psychometric data. Variables were added into regression models in a forward, stepwise manner, and Fischer's statistic was used to prove significance of models. Significance was set at *p* < 0.05.

## Results

### Sample Characteristics

Sample characteristics are detailed in Table 2. There was a strong difference in BMI z-scores between the groups, with the OBE classified on average as obese and the NW as normal weight.

**Table 2:** Sample characteristics.

	**OBE T0 (*n* = 60)**	**OBE T1 (*n* = 53)**	**NW (*n* = 27)**	**FDR**	**FDR**
				**OBE T0 vs. NW**	**OBE T0 vs. T1**
Sex (♂:♀)	28:32	23:30	15:12	n.s.	n.a.
Age (years)	13.03 ± 1.89	13.04 ± 1.85	12.5 ± 0.9	n.s.	n.a.
[Min–max]	[9–17]	[9–17]	[11–14]		
BMI z-score	2.5 ± 0.6	2.3 ± 0.6	−0.2 ± 0.6	<0.001^***^	<0.001^***^
[Min–max]	[1.1–3.7]	[0.6–3.6]	[−1.3–1.1]		
Body fat %	34.33 ± 4.93	30.59 ± 4.31	15.04 ± 6.94	<0.001^***^	<0.001^***^
[Min–max]	[21–45]	[19–40]	[5–28]		

*Values are presented as mean ± standard deviation. Statistical significance was determined via adjusted p-values using the false discovery rate (FDR) method*.

*** *indicates FDR < 0.05*.

Values are presented as mean ± standard deviation. Statistical significance was determined via adjusted p-values using the false discovery rate (FDR) method. ^*^FDR < 0.05.

### Cross-Sectional Differences in Sleep Measures According to Weight Status

Between-group comparison data are presented in Table 3. OBE and NW differed significantly for all parent-reported questionnaires, with OBE consistently reporting poorer sleep outcomes (CSHQ: z = −2.61, FDR = 0.018; ESS-E: z = −2.33, FDR = 0.027; PSQ-SRBD: z = −4.87, FDR <0.001), but not in the self-reported questionnaires. Differences in parent-reported sleep duration were not significant; however, the difference in self-reported sleep duration of the children was significant (FDR <0.001), with OBE reporting less average hours of sleep (five-point scale where 5 = 9–11 h down to 1 = <5 h). When further assessed by questionnaire subscales, no statistical differences between groups were observed.

**Table 3:** Sleep results comparison between OBE and NW.

	**OBE T0 (*n* = 60)**	**NW (*n* = 27)**	**Test**	**FDR**
**Self-reported assessments**				
SSR	5.67 ± 1.88	5.19 ± 1.77	t(84) = 1.09	0.933
PDSS	10.13 ± 5.56	11.04 ± 4.22	t(84) = −0.74	0.253
Sleep duration	3.59 ± 0.13	4.52 ± 0.15	z = −4.02	<0.001^***^
Sleep quality	1.58 ± 1.75	1.22 ± 1.02	z = −0.08	0.933
**Parent-reported assessments**				
Sleep duration	8.62 ± 1.28	8.58 ± 0.70	z = −0.34	0.732
CSHQ total	43.62 ± 8.26	39.89 ± 5.19	z = −2.61	0.018^*^
Bedtime resistance	7.26 ± 1.90	6.57 ± 1.04	z = −1.80	0.192
Sleep-onset delay	1.41 ± 0.63	1.30 ± 0.56	z = −0.66	0.510
Sleep duration	4.59 ± 1.79	3.75 ± 1.07	z = −1.84	0.192
Sleep anxiety	4.85 ± 1.43	4.36 ± 0.79	z = −1.59	0.192
Night wakings	3.41 ± 0.73	3.22 ± 0.52	z = −1.12	0.348
Parasomnias	8.28 ± 1.36	7.73 ± 0.94	z = −1.55	0.192
Sleep-disordered breathing	3.84 ± 1.05	3.33 ± 0.70	z = −2.39	0.133
Daytime sleepiness	12.82 ± 3.34	12.09 ± 2.56	z = −0.88	0.432
ESS-E total	4.85 ± 3.62	2.75 ± 2.31	z = −2.33	0.027^*^
PSQ-SRBD total	0.19 ± 0.13	0.06 ± 0.07	z = −4.87	<0.001^***^
Snoring	0.25 ± 0.34	0.10 ± 0.29	z = −2.28	0.068
Sleepiness	0.15 ± 0.22	0.09 ± 0.17	z = −0.85	0.396
Behavior	0.13 ± 0.19	0.04 ± 0.09	z = −1.84	0.099

*Values are presented as mean ± standard deviation. Statistical significance was determined via adjusted p-values using the false discovery rate (FDR) method*.

*** *indicates FDR <0.001*,

* *indicates FDR <0.05. CSHQ, children's sleep habits questionnaire; ESS-E, Epworth sleepiness scale for parents; PDSS, pediatric daytime sleepiness scale; PSQ-SRBD, pediatric sleep habits questionnaire: sleep-related breathing disorder; SSR, sleep self-report*.

### Longitudinal Changes in Sleep Measures of Children With Obesity During Inpatient Therapy

The interval between time points T0 and T1 had an average of 26.4 days (SD 8.2; min–max: 14–57). Positive results were achieved during inpatient therapy for the physical outcomes, with a mean decrease in BMI z-score of 0.21 (min–max: 0.0–0.6; FDR <0.001) and body fat percentage of 3.80% (min–max: 0–9%; FDR <0.001) (Table 2).

Sleep outcomes remained unchanged during inpatient therapy (Table 4). The children reported a reduction in sleep quality. Spearman's rank correlation matrix showed no significant correlations between changes in children's reported sleep outcomes and anthropometric changes during the treatment period (Supplementary Table 1).

**Table 4:** Comparison between pre- and post-intervention.

	**Pre-intervention (T0; *n* = 60)**	**Post-intervention (T1; *n* = 53)**	**Change from T0 to T1 (*n* = 53)**	**Test**	**FDR**
SSR	5.70 ± 1.88	5.70 ± 1.89	0.19 ± 3.19	t(52) = 0.00	1.000
PDSS	10.79 ± 5.42	11.06 ± 5.89	0.26 ± 3.45	t(52) = −0.56	1.000
Sleep duration	3.60 ± 0.91	3.60 ± 0.98	0.13 ± 1.67	z = −0.05	1.000
Sleep quality	1.52 ± 1.68	3.82 ± 2.85	2.30 ± 2.79	z = −4.82	< 0.001^***^

*Values are presented as mean ± standard deviation. Statistical significance was determined via adjusted p-values using the false discovery rate (FDR) method*.

*** *indicates FDR <0.001. PDSS, pediatric daytime sleepiness scale; SSR, sleep self-report*.

### Prediction of Sleep Behaviors by Psychological Stress Factors

Regression analyses were conducted to assess the predictive power of the baseline psychological factors on baseline sleep measures for OBE and NW together (Table 5). Prior to the regression procedure, we calculated a Spearman correlation matrix and corrected for multiple comparisons using the FDR method (see Methods). Only variables that remained significant were included in the regression analyses. This procedure resulted in a limited number of predictors that entered the regression: for two of six models, only three incoming predictors were selected; one analysis was conducted with 2, one with 4, one with 9, and the last with 12 predictors. Variables that were preselected for each modeling procedure are presented in Supplementary Table 2. Higher anxiety (STAI-C) and destructive-anger-related emotion regulation (SSKJ subscale) scores were moderate predictors of self-reported poorer sleep scores (SSR, R^2^ = 0.277). Destructive-anger-related emotion regulation and female gender were positively related to the increased self-reported daytime sleepiness (PDSS, R^2^ = 0.202). None of the psychological variables but age contributed to the parent-reported sleep duration with older children having a shorter sleep time period (R^2^ = 0.222). Parent-reported child's quality of life (ILK) was a moderate predictor of poorer parent-reported sleep scores (CSHQ, R^2^ = 0.14) and parent-reported daytime sleepiness (ESS-E, R^2^ = 0.202). In addition, higher self-reported somatic symptom scores also predicted parent-reported daytime sleepiness (ESS-E, R^2^ = 0.202).

**Table 5:** Summary of regression models.

**Predictor**	**ß**	***F***	**Significance**	**Importance**	**R^**2**^**
	Sleep self-report (SSR)				0,277
Destructive-anger-related emotion regulation^b^	0.059	14,871	<0.001	0,618	
Anxiety symptoms^a^	0.161	9.197	0.003	0.382	
	Pediatric daytime sleepiness scale				0.202
Destructive-anger-related emotion regulation^b^	0.068	11.894	0.001	0.562	
Gender (f)	3.024	9.287	0.003	0.438	
	Sleep duration (parent reported)				0.222
Age	−0.268	25.494	<0.001	1.0	
	Children's sleep habits questionnaire				0,14
Parent-reported quality of life^d^	−0.599	15.009	<0.001	1.000	
	Epworth sleepiness scale				0.202
Parent-reported quality of life^d^	−0.294	16.785	<0.001	0.773	
Somatization^c^	0.065	4.928	0.029	0.227	
	Pediatrics sleep habits questionnaire				0,16
OBE group	0.109	17.350	<0.001	1.000	

*Psychological stress and eating behavior parameters measured via the following questionnaires:*

a *STAI-C;*

b *SSKJ;*

c *I-KJ;*

d *ILK*.

Importantly, the variable group (OBE versus NW) did not contribute to any of the above-described models. In contrast, being in the intervention group (therefore dependent on weight status) was a moderate predictor for higher parent-reported sleep-disordered breathing (PSQ-SRBD, R^2^ = 0.16).

## Discussion

This study aimed to investigate the interrelation of sleep and psychological factors in children and adolescents with obesity in an inpatient weight-loss treatment environment. The study involved a combined approach including cross-sectional (comparison between OBE and NW) and longitudinal design aspects (comparison of OBE over time).

Sleep behaviors were measured through multiple validated questionnaires for pediatric populations. The OBE children and adolescents had more unfavorable sleep scores than the NW for the three parent-reported sleep questionnaires (CSHQ, ESS-E, and PSQ-SRBD), thereby supporting our hypothesis that less favorable sleep behaviors would be observed in the OBE group; however, no significant difference was found for the child-reported questionnaires. These results were in line with the similarly focused study by Beebe et al. (4), where they also reported poorer parent-reported sleep outcomes (CSHQ and PSQ-SRBD) in children and adolescents with obesity. It was additionally hypothesized that sleep duration would be shorter in OBE than NW; however, this was only partially confirmed as a significant difference was only apparent for the child-reported sleep duration, with OBE reporting an average sleep duration of one point lower on the five-point scale (equating to approximately 1 h less) than NW. This discrepancy between parent- and child-reported differences was also found by Beebe et al., but with parent-reported sleep duration differing between groups (4). These discrepancies were not unexpected as children have a different perception of many dimensions, including their own health and the duration of time, due to the developmental process and a different understanding of temporal information, which can lead to misinterpretation and miscommunication of information (49). However, this discrepancy between self-reported and parent-reported data decreases with the age of the child (49). The discrepancy can also be due to the fact that personal habits such as snoring or restless sleep may be better assessed by observers than by oneself. For this reason, the collection of information from caregivers and family members is mandatory in the diagnosis of sleep disorders (50).

The second hypothesis that sleep outcomes in OBE will improve over the course of the inpatient treatment was not confirmed in the present study, which is to our knowledge the first longitudinal inpatient study with this focus. The self-reported sleep outcomes of the OBE children did not differ between the beginning and end of therapy. The self-reported sleep quality decreased during inpatient treatment, which was unsurprising due to the unfamiliar sleeping environment and impact of the inpatient therapy consisting of stricter sleep and day routines. Additionally, it would be expected that the OBE group would have similar sleep measures during treatment because of this structure, therefore acting as a constant variable during this time.

It was also hypothesized that improvements in sleep measures would be associated with improvements in BMI z-score and body fat percentage. Statistically and clinically significant changes in BMI z-score and body fat percentage were achieved during the inpatient weight-loss program; however, these positive changes were not correlated with changes in sleep outcomes. The average interval between T0 and T1 was 26 days, which although sufficient to achieve significant anthropometric changes may be too short to notice significant changes in sleep behavior. Studies with longer timeframes have reported improvements in sleep parameters, in particular in relation to sleep-disordered breathing and obstructive sleep apnea, in children and adolescents following multidisciplinary weight-loss interventions (51–53). Roche et al. reported improved sleep duration and sleep architecture after a 9-month weight-loss program in adolescents with sleep apnea which also achieved improvements in body weight and body composition (53). This implies that continued weight-loss therapy after inpatient treatment may continue to benefit both anthropometric and sleep outcomes in children and adolescents with obesity.

The third hypothesis that unfavorable sleep behaviors in children and adolescents are related to psychological factors (anxiety, stress coping strategies, and depression), quality of life, and weight status was partially confirmed only for parent reported “snoring.” Higher anxiety and destructive-anger-related emotion regulation were associated with poorer child-reported sleep and increased daytime sleepiness. These same sleep outcomes as assessed by parent questionnaires were associated with parent-reported quality of life status, with worsened quality of life predicting poorer sleep values. Importantly, group (OBE versus NW) was the only significant variable for sleep-disordered breathing (PSQ-SRBD), which has established associations with carrying excess weight in adults (54) and children (4, 26, 52). On the other hand, the weight status did not significantly contribute to any other model exploring the relationship between psychological variables and sleep measures. These results raise the question whether or not psychological factors have a greater impact on sleep behavior than the influence of excess body weight. Little is known about the predictive nature of psychological factors and quality of life on sleep in pediatric populations, although correlations have been reported in cross-sectional studies (4, 55, 56). These findings are reflected in more serious cases in the Diagnostic and Statistical Manual for Mental Disorders (DSM-5) which lists sleep disturbance as a criterion for diagnosis of multiple psychological disorders including major depression and generalized anxiety disorder (57). Therefore, the inclusion of psychological elements in pediatric weight-loss therapy may have extended benefits onto improving sleep outcomes.

A particular strength of the present study is that it assessed a pediatric population with obesity in an inpatient setting through both cross-sectional and longitudinal approaches. Data on inpatient subpopulations in childhood obesity research are scarce, and especially so for data on sleep and psychological factors. The methods used for data collection included questionnaires (validated for the age group and language) for sleep and psychological measures, allowing for practical, minimally invasive, and easily comparable data on sleep behaviors.

Although the questionnaires included have been validated with the appropriate target populations, the potential biases relating to social desirability or inability to introspect, especially in children and in parent populations, cannot be ruled out (49, 58). However, the impact of this was further mitigated through the inclusion of parent-reported questionnaires. One limitation may be that objective sleep measures, including sleep laboratories, polysomnography, or actigraphy, were not included. Finally, data were collected for two settings (home and inpatient stay); the range in duration for inpatient therapy (16 to 70 days) and the age range (9 to 17 years) were wide, resulting in heterogeneity for intervention group data. Because of the small sample size, the results of regression analysis should be interpreted only as preliminary and the one that indicates important factors for the future studies with increased sample size.

## Conclusion

This study aimed to assess sleep behavior in relation to a variety of psychological factors in attempts to further understand the impact of childhood obesity on sleep and its possible role in inpatient weight-loss treatment for children and adolescents. Overall, we showed that children and adolescents with the indication for inpatient weight-loss treatment have shorter sleep duration and are more impacted by poor sleep behaviors, daytime sleepiness, and sleep-disordered breathing than their normal-weight peers. These factors were also correlated with multiple psychological assessments, including depression and anxiety symptoms, poorer quality of life, and destructive-anger-related emotion regulation. These results show that addressing sleep behaviors, through sleep hygiene education, as part of weight-loss therapy may provide both physical and psychological benefits to children and adolescents with obesity. More research is needed on the relationship between sleep and psychological factors, and whether they can be integrated clinically in prevention and management of childhood obesity and possibly also other pediatric diseases.

## Data Availability Statement

The raw data supporting the conclusions of this article will be made available by the authors, without undue reservation.

## Ethics Statement

The studies involving human participants were reviewed and approved by Ethics Committee of the medical faculty for the University Tübingen, Germany. Written informed consent to participate in this study was provided by the participants' legal guardian/next of kin.

## Author Contributions

JC and NM were responsible for the data analysis and interpretation and drafted the paper. IM was responsible for the conception, funding, design and preparation of the study, data analysis, and data interpretation. AW analyzed the data. SZ and PE were responsible for the conception and design of the study. YR, FJ, and MU were involved in the data interpretation. All authors revised the manuscript and approved the final version.

## Conflict of Interest

The authors declare that the research was conducted in the absence of any commercial or financial relationships that could be construed as a potential conflict of interest.
