# Supplementary material for: Impact of Childhood Obesity and Psychological Factors on Sleep
Source: Front Psychiatry. 2021 Jul 9;12:657322. doi: 10.3389/fpsyt.2021.657322 (PMC8298750; doi:10.3389/fpsyt.2021.657322)
Supplement: Supplementary file 1 [file Table_1.DOCX]

**Table 1 Supp.** Spearman’s rank correlation matrix between changes in sleep outcomes and anthropometric changes during the treatment period.

|  | ∆ SSR | ∆ PDSS | ∆ Sleep duration | ∆ Sleep quality |
| --- | --- | --- | --- | --- |
| ∆ BMI z-score | 0,085  (p=.458) | 0,184  (p=.105) | 0,141  (p=.325) | 0,053  (p=.718) |
| ∆ Percentage body fat | -0,21  (p=.139) | 0,072  (p=.618) | -0,028  (p=.851) | -0,142  (p=.341) |

Data are presented as correlation coefficient (two-tailed significance); SSR – Sleep Self Report Questionnaire; PDSS – Pediatric Daytime Sleepiness Scale
